# Stachydrine is effective and selective against blast phase chronic myeloid leukaemia through inhibition of multiple receptor tyrosine kinases

**DOI:** 10.1080/13880209.2022.2044862

**Published:** 2022-03-29

**Authors:** Ruixin Gu, Wei Zhang, Dandan Xu

**Affiliations:** a Department of Traditional Chinese Medicine, Puai Hospital, Tongji Medical College, Huazhong University of Science and Technology, Wuhan, China; b Public Health Division, Hospital of Huazhong Agricultural University, Wuhan, China; c Department of Rehabilitation Medicine, Hubei Provincial Hospital of Traditional Chinese Medicine Affiliated to Hubei University of Traditional Chinese Medicine, Wuhan, China

**Keywords:** Leukaemia, synergy, traditional Chinese medicine

## Abstract

**Context:**

Resistance to BCR-ABL tyrosine kinase inhibitor (TKI) is the cause of treatment failure in blast phase chronic myeloid leukaemia (BP-CML). Agents that act synergistically with BCR-ABL TKI are required to improve response.

**Objective:**

This work investigated the effects of stachydrine in CML.

**Materials and methods:**

CML cells were treated with control or stachydrine at 20, 40 and 80 µM. Proliferation and apoptosis were examined after 72 h treatment. Combination studies were performed in four groups: control, TKI, stachydrine and the combination of stachydrine and TKI. Immunoblotting analysis was performed in CML cells after 24 h treatment.

**Results:**

Stachydrine inhibited K562 (IC_50_ 61 µM), KCL22 (IC_50_ 141 µM), LAMA84 (IC_50_ 86 µM), Ba/F3 T315I (IC_50_ 26 µM), Ba/F3 WT (IC_50_ 22 µM) and KU812 (IC_50_ 35 µM) proliferation, and induced apoptosis in these CML cell lines. Stachydrine significantly induced apoptosis, inhibited colony formation and self-renewal in BP-CML CD34^+^ cells. The combination index of stachydrine and TKI combination was <1. Compared to TKI alone, the combination of stachydrine and TKI significantly induced more apoptosis and decreased colony formation in BP-CML CD34^+^ cells. Stachydrine decreased phosphorylation levels of multiple receptor tyrosine kinases in CML cells.

**Discussion and conclusions:**

Our study is the first to demonstrate (1) the anticancer activity of stachydrine on primary patient cancer cells; (2) the inhibitory effects of stachydrine on cancer stem cells; (3) the synergism between stachydrine and other anticancer drugs.

## Introduction

Chronic myeloid leukaemia (CML) is a haematopoietic malignancy caused by the presence of the fusion gene BCR-ABL in stem/progenitor cells (Sawyers 1999). BCR-ABL encodes a constitutively active tyrosine kinase that leads to upregulation of signal transduction pathways involved in cell survival and growth, such as Ras/MEK/MAPK and PI3K/AKT (Ren 2005). The introduction of BCR-ABL tyrosine kinase inhibitors (TKIs, e.g., imatinib, dasatinib and ponatinib) that bind to the ATP-binding site of Abl, has led to a remarkable clinical response to treat CML. However, patients develop resistance to TKIs and progress to blast phase (BP). Mutations in the Abl kinase domain and Bcr-Abl protein overexpression are the main mechanisms that contribute to TKI resistance (Perrotti et al. 2010; Corbin et al. 2011). Therefore, identification of agents that overcome TKI resistance is needed to improve clinical response in blast phase chronic myeloid leukaemia (BP-CML) patients.

*Leonurus japonicus* Houtt (Labiatae, commonly named Chinese motherwort) is classified as the highest grade of non-toxic traditional Chinese medicine (TCM) in the Sheng Nong’s herbal classic literature. It is mainly used for treatment of menoxenia, dysmenorrhoea and amenorrhoea in women due to its blood circulation stimulating effects (Shang et al. 2014). Stachydrine is (2*S*)-1,1-dimethylpyrrolidine-2-carboxylic acid and is one of the major bioactive ingredients of *Leonurus japonicus*. Stachydrine displays bioactivities for treating uterine and cardiovascular diseases (Cheng et al. 2020). Recent studies revealed that stachydrine exerts anticancer effects. Stachydrine suppresses viability and migration of astrocytoma cells (Liu et al. 2018). It inhibits proliferation and induces apoptosis of breast and prostate cancer cells (Rathee et al. 2012; Wang et al. 2017). The molecular mechanisms of stachydrine’s action in cancer are via inhibiting multiple oncogenic pathways, including Akt/Erk and CXC chemokine receptor 4 (CXCR4). However, little is known on the effects of stachydrine in leukaemia.

In this study, we systematically (1) evaluated the *in vitro* efficacy of stachydrine on CML cell lines and stem/progenitor cells from BP-CML patients; (2) determined the combinatory effects of stachydrine and BCR-ABL TKI; (3) investigated the selective activity of stachydrine using normal cord blood (CB) cells; (4) analysed the underlying mechanisms of stachydrine focussing on receptor tyrosine kinase (TRK) pathways.

## Materials and methods

### BP-CML samples, CD34^+^ isolation and CML cell lines

This study was approved by the ethics committee of Tongji Medical College, Huazhong University of Science and Technology (approval ID: 2016-068) and conducted in accordance with the Declaration of Helsinki. Primary BP-CML peripheral blood or bone marrow and normal CB were obtained from patients at the Puai Hospital with written informed consent approved by the institutional review board. CB or BP-CML CD34^+^ cells were isolated using the same protocol described in our previous study (Gu et al. 2018). CD34^+^ cells were cultured in serum free-StemPro complete medium (Life Technologies, Carlsbad, CA) supplemented with stem cell factor, granulocyte-macrophage colony-stimulating factor and macrophage inflammatory protein-1α at 100 pg/mL each; granulocyte colony-stimulating factor and interleukin 6 at 1000 pg/mL and leukaemia inhibitory factor at 50 pg/mL.

### CML cell line, compounds, Western blotting and antibodies

Human CML cell lines (KCL22, KU812, K562 and LAMA84) were obtained from American Type Culture Collection (Manassas, VA). Two murine CML cell lines Ba/F3-BCR-ABL (Ba/F3 WT) and Ba/F3-BCR-ABL-T315I (Ba/F3 T315I) were obtained from Creative Biogene Biotechnology (Shirley, NY). All cell lines were cultured in RPMI1640 medium containing foetal bovine serum (FBS) with a final concentration of 10% and kept at 37 °C, 5% CO_2_ atmosphere. Imatinib, ponatinib and stachydrine were obtained from Selleckchem Inc. (Houston, TX). The purities of imatinib, ponatinib and stachydrine were 99.84%, 99.79% and 98.86% respectively according to manufacturer’s HPLC data. Imatinib was reconstituted in PBS. Ponatinib and stachydrine were reconstituted in dimethyl sulphoxide (DMSO). All compounds were stored in −20 °C as individual aliquots. Western blotting was performed after 24 h drug treatment using standard protocol. Antibodies against p-Bcr-Abl (Tyr177), Bcr-Abl, p-Crkl (Tyr207), Crkl, p-Src (Tyr416), Src, p-c-Kit (Tyr823), c-Kit, p-Stat3 (Tyr705), Stat3 and β-actin were obtained from Cell Signaling (Boston, MA).

### Flow cytometry

Cells (10^5^ per well) were plated in each well within a 12-well plate. After 72 h drug treatment, cells were harvested for staining using Annexin V-FITC/7-AAD (Beckman Coulter, Brea, CA). Apoptotic cells were analysed using flow cytometry on Beckman FC500. Annexin V percentage was quantified using CXP software (Beckman Coulter, Brea, CA).

### BrdU proliferation and combination index (CI)

Cells (5000 per well) were plated to each well in a 96-well plate. After 72 h drug treatment, cell proliferation activities were evaluated by BrdU proliferation assay kit (Abcam, Cambridge, UK). For combination studies, the IC_50_ of each drug was first determined. The cells were then treated with increasing doses of single drug alone or an equipotent constant-ratio combination of both drugs. After 72 h drug treatment, proliferation activity was determined. The CI was calculated using the CalcuSyn software (Biosoft, St. Louis, MO). If CI is less than 1, the combination is synergistic; if CI is equal to 1, the combination is additive; if CI is more than 1, the combination is antagonistic (Huang et al. 2017).

### Colony-forming and serial-replating assays

Cell suspension (100 μL) containing 2000 cells, together with various concentrations of drugs and 1000 μL of HSC-CFU complete methylcellulose medium (Miltenyi Biotec, Bergisch Gladbach, Germany) were plated onto a 35 mm Petri dish and incubated in 37 °C, 5% CO_2_ atmosphere. After 2 weeks incubation, colonies were formed and counted under the microscope. To examine the self-renewal ability of cells, serial-replating assay was performed by picking up individual colonies formed in colony-forming assay and mixing with 100 μL of HSC-CFU complete methylcellulose. The mixture was plated into a 96-well plate and incubated in 37 °C, 5% CO_2_ atmosphere. After 2 weeks incubation, serial replating capacity is determined by calculating the percentage of number of wells with colonies formed among total number of wells with colonies plated.

### Statistical analyses

All experiments were repeated three times. The data were expressed as mean ± SD. A one-way analysis of variance (ANOVA) was performed on groups of different drug conditions. To ascertain that the combinatory drug conditions were indeed efficacious, we used a single tail Student’s *t*-test on TKI and combinatory conditions. For testing of dosages, all comparisons were made with its respective control conditions only (two categorical variables) and hence used Student’s *t*-test. An unpaired Student’s *t*-test was applied to determine statistical significance with *p* < 0.05.

## Results

### Stachydrine is active against imatinib-sensitive and imatinib-resistant CML cell lines

**Figure 1.**
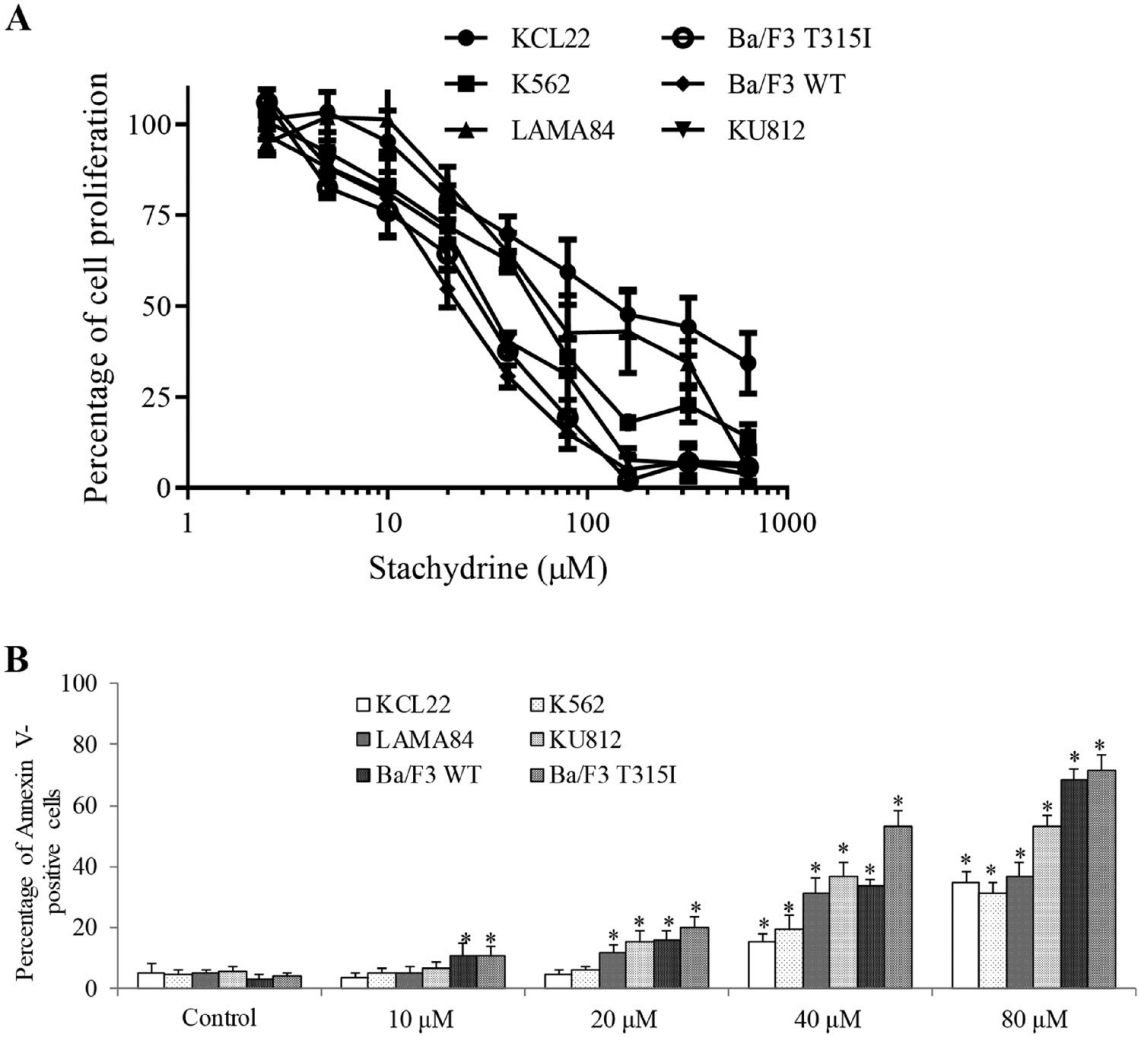
Stachydrine is active against a panel of CML cell lines. Stachydrine significantly decreases proliferation (A) and increases apoptosis (B) of CML cells. Results shown were relative to control (set as 100%) in proliferation assay. KCL22, K562, LAMA84 and KU812 are human CML cell lines. Stachydrine concentrations at 2.5, 5, 10, 20, 40, 80, 160, 320 and 640 µM were tested in proliferation assay. Ba/F3 WT and Ba/F3 T315I are murine cell lines transfected with Bcr-Abl (WT) and Bcr-Abl T315I. *p < 0.05, compared to control.

The anti-proliferative and pro-apoptotic effects of stachydrine on CML cells were determined using BrdU and Annexin V labelling, respectively. A panel of CML cell lines that represented diverse cellular origins and genetic profiles were selected to demonstrate the biological activities of stachydrine, including imatinib-sensitive (KCL22, KU812, LAMA84, K562 and Ba/F3 WT) and imatinib-resistant (Ba/F3 T315I) cell lines. We found that stachydrine inhibited proliferation of all tested CML cell lines (Figure 1(A)). The IC_50_ of stachydrine is shown in Table 1. In the Bcr-Abl T315I-harbouring cell line Ba/F3 T315I, the IC_50_ for stachydrine was only 1.2-fold higher than in its Brc-ABL WT Ba/F3 cell line. Among human CML cell lines, KCL22 was the most resistant with IC_50_ at ∼141 μM and KU812 was the most sensitive with IC_50_ at ∼35 μM to stachydrine. In addition, stachydrine significantly induced apoptosis of CML cell lines (Figure 1(B) and Figure S1). Notably, stachydrine at 80 µM induced up to 80% apoptosis in imatinib-resistant cell line Ba/F3 T315I similar to Ba/F3 WT, suggesting that imatinib-resistant CML cells do not exhibit cross-resistance to stachydrine.

**Table 1.** The IC_50_ of stachydrine on CML cell lines.

Cell line	Stachydrine IC_50_, mean ± SD, μM
KCL22	141 ± 16
K562	61 ± 5
LAMA84	86 ± 11
KU812	35 ± 5
Ba/F3 WT	22 ± 2
Ba/F3 T315I	26 ± 1

IC_50_ values were determined using Prism software. Stachydrine concentrations at 2.5, 5, 10, 20, 40, 80, 160, 320 and 640 µM were tested in proliferation assay.

### Stachydrine is active against BP-CML stem/progenitor cells

**Figure 2.**
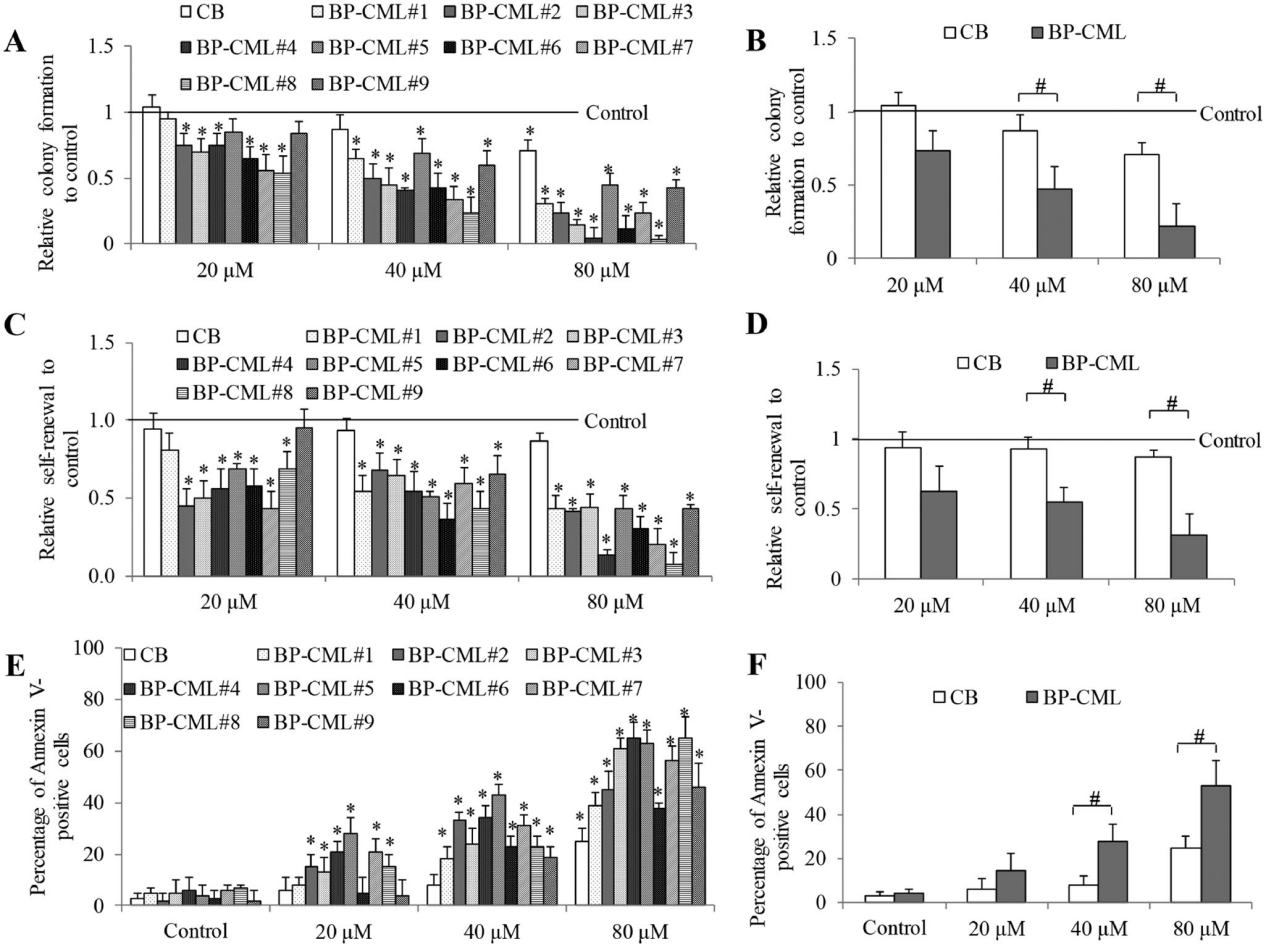
Stachydrine is active against CML stem/progenitor cells. The inhibitory effects of stachydrine on colony formation (A, B) and self-renewal capacity (C, D) of BP-CML stem/progenitor cells. (E, F) The stimulatory effect of stachydrine on apoptosis of BP-CML stem/progenitor cells. Individual (A, C and E) and average (B, D and F) values of BP-CML (*n* = 9) are shown. CB value was the average of six samples. Results shown were relative to control (set as 1) in colony-forming and serial-replating assays. CD34^+^ cells from CB and BP-CML were used for all assays. ^#^*p* < 0.05, comparison between CB and CP-CML. *p < 0.05, compared to control.

Having shown the inhibitory effects of stachydrine in imatinib-resistant CML cells, we next evaluated its performance on primary stem/progenitor cells from treatment-naïve and -resistant BP-CML patients. To mimic the *in vivo* experimental conditions, CD34^+^ cells were purified from nine patients and maintained in culturing medium supplemented with cytokines and growth factors similar to that found in stroma-conditioned medium from bone marrow (Chu et al. 2004). The patients’ clinical information is summarized in Table 2. Many patients selected in our study had Bcr-Abl mutations, such as E255K, T315I and E253K, and were TKI-resistant. To evaluate whether stachydrine displays anti-selective leukaemia activity, CD34^+^ cells from normal CB are used as normal control. We performed colony-forming and serial replating assays to examine whether stachydrine affected proliferation, differentiation and self-renewal capacity of BP-CML and CB stem/progenitor cells. Although with varying efficacy in different patient samples, stachydrine significantly inhibited colony-forming and self-renewal capacities of BP-CML CD34^+^ cells (Figure 2(A–D) and Figure S2). Stachydrine at the same concentrations also inhibited colony-forming and self-renewal capacity of CB CD34^+^ cells, but to a lesser extent than in BP-CML counterparts. Stachydrine was also more effective in inducing apoptosis in BP-CML than CB CD34^+^ cells (Figure 2(E,F) and Figure S3). In addition, we did not observe any associations between the efficacy and BP-CML patients’ clinicopathological characteristics, such as Bcr-Abl mutation, overexpression and TKI-resistance (Table 2).

**Table 2.** BP-CML patients’ information.

Patient number	BCR-ABL1 mutation	BCR-ABL1 overexpression	TKI-resistant
BP-CML#1	E255K	NA	Newly diagnosed
BP-CML#2	T315I	No	Ponatinib
BP-CML#3	None	Yes	Imatinib
BP-CML#4	E255V	No	Imatinib, dasatinib
BP-CML#5	None	No	Imatinib, dasatinib
BP-CML#6	T315I	No	Newly diagnosed
BP-CML#7	T315I	No	Newly diagnosed
BP-CML#8	E453K	Yes	Imatinib
BP-CML#9	T315I	NA	Newly diagnosed

### The combination of stachydrine and Bcr-Abl TKIs is synergistic against CML

**Figure 3.**
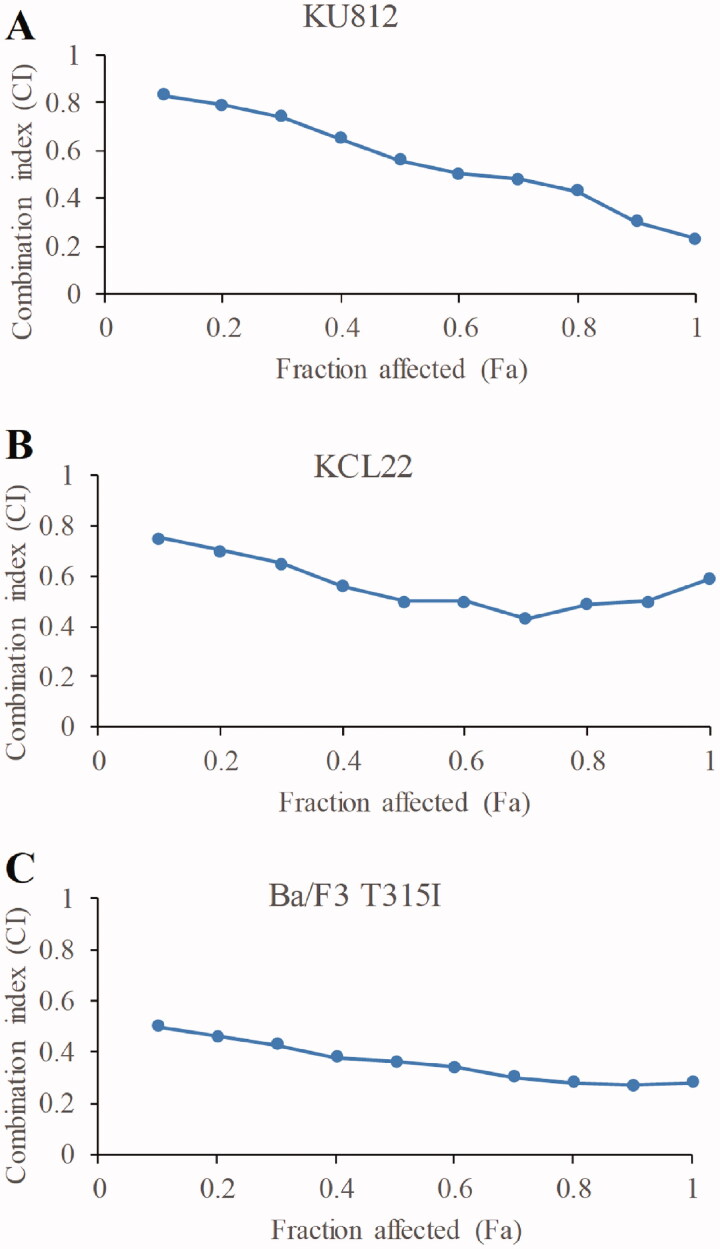
The combination of stachydrine and Bcr-Abl TKIs are synergistic in CML cells. The combination index (CI) vs. fraction affected plot showing that combination of stachydrine and Bcr-Abl TKIs is synergistic in inhibiting proliferation of KU812 (A), KCL22 (B) and Ba/F3 T315I (C) cells. Imatinib was used for KU812 and KCL22 cells and ponatinib was used in Ba/F3 T315I cells. If CI is less than 1, the combination is synergistic; if CI is equal to 1, the combination is additive; if CI is more than 1, the combination is antagonistic.

To investigate the translational potential of stachydrine in CML, we further evaluated its combinatory efficacy with Bcr-Abl TKIs by performing combination studies. Based on Chou and Talalay’s method (Chou 2010), we treated CML cell lines with single drug alone or both drugs at an equipotent constant ratio. After drug treatment, we measured proliferation activity level in each treatment setting and calculated its CI. We used imatinib and ponatinib as TKIs for imatinib-sensitive and imatinib-resistant lines, respectively. The IC_50_ of TKIs for CML cell lines is shown in Table 3. Isobologram analysis results are shown in Figure 3. Synergy between stachydrine and TKI which was defined as CI <1 (Huang et al. 2017), was seen in KCL22, KU812 and Ba/F3 T315I cell lines.

**Table 3.** The IC_50_ of TKIs on CML cell lines.

Cell line	TKI IC_50_, mean ± SD, nM
KCL22	83 ± 3 (imatinib)
KU812	51 ± 5 (imatinib)
Ba/F3 T315I	5 ± 0.6 (ponatinib)

IC_50_ values were determined using Prism software. Imatinib concentrations at 5, 10, 25, 50, 100, 200 and 400 nM were tested in proliferation assay. Ponatinib concentrations at 0.1, 0.2, 0.5, 1, 2, 4, 8, 16 and 32 nM were tested in proliferation assay. Imatinib was used for KCL22 and KU812. Ponatinib was used for Ba/F3 T315I.

**Figure 4.**
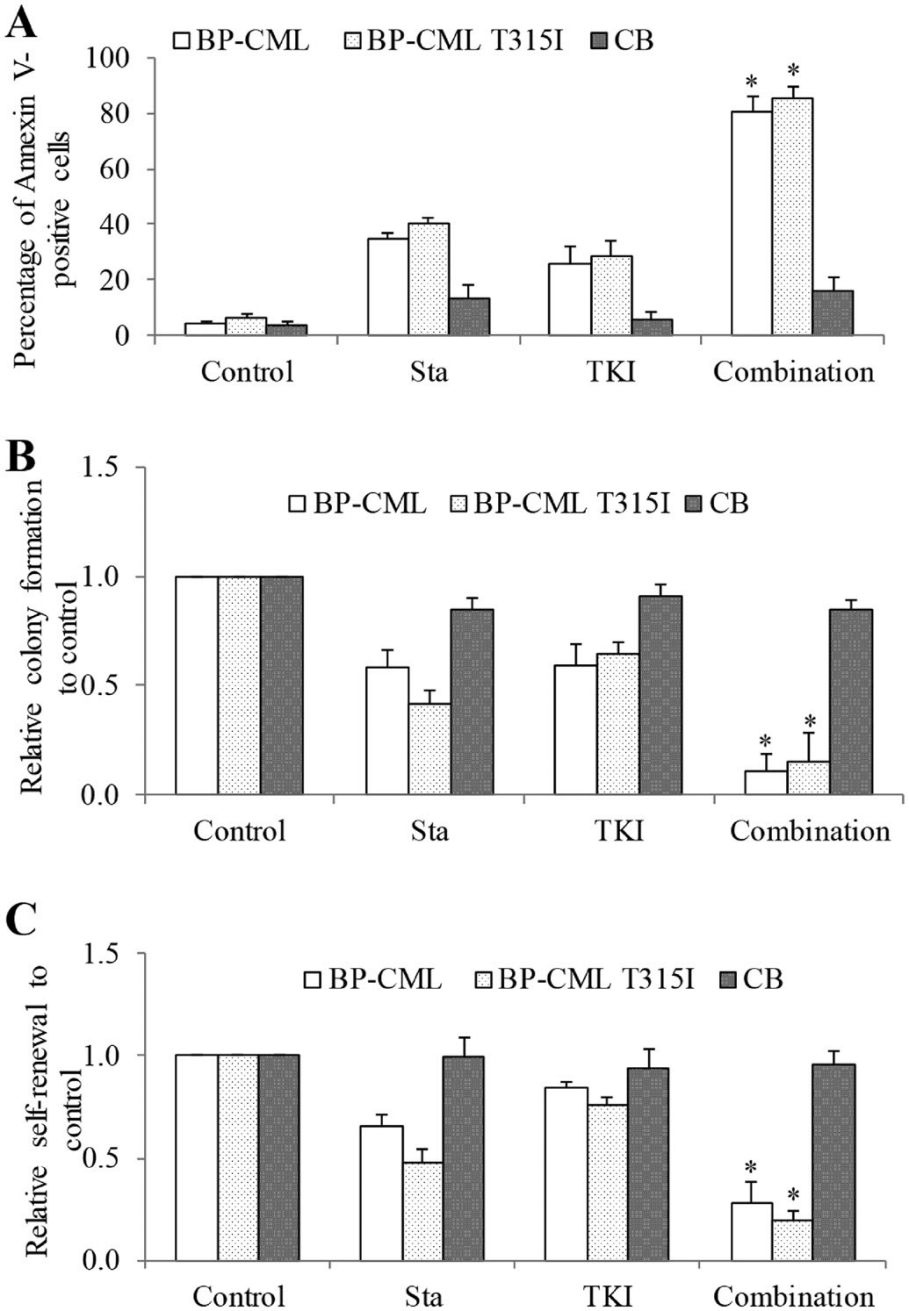
Stachydrine significantly enhances efficacy of Bcr-Abl TKIs in BP-CML CD34^+^ cells. The combination of stachydrine with Bcr-Abl TKIs is significantly superior to TKIs alone in inducing apoptosis (D), inhibiting colony formation (E) and suppressing self-renewal (F) of BP-CML and BP-CML T315I but not CB CD34^+^ cells. Stachydrine at 30 μM, dasatinib (for BP-CML) at 100 nM, ponatinib (for BP-CML T315I) at 50 nM were used. Graphs presented are mean of the results obtained from five patient-derived CML samples. *p < 0.05, compared to TKI alone.

We next performed combination studies using stachydrine and TKI on BP-CML CD34^+^ cells. In order to observe the combinatory effects of stachydrine and TKI in CML stem/progenitor cells, we used the concentration of TKI that induces <40% apoptosis, and inhibits <40% colony formation and self-renewal in CML stem/progenitor cells as single drug alone: 100 nM of dasatinib and 50 nM ponatinib (Table 4). ANOVA showed significant difference among different drug conditions (*p* value <0.001). Consistent with CML cell lines, treatment of BP-CML CD34^+^ cells with the combination of stachydrine and TKI resulted in remarkable induction of apoptosis, and inhibition of colony formation and self-renewal, whereas only modest induction of apoptosis and reduction in inhibition of colony formation and self-renewal were seen with TKI alone (Figure 4(A–C)). Of note, the combination was much more effective than TKI alone in BP-CML T351I samples. In contrast, treatment with the combination of stachydrine and TKI did not result in further significant inhibition of survival, colony formation and self-renewal in normal CB CD34^+^ cells, compared to TKI alone. These results demonstrate that the combination is synergistic and selective against BP-CML CD34^+^ cells.

**Table 4.** The inhibitory effects of TKI on BP-CML CD34^+^ cells.

	Concentration (nM)	Annexin V percentage	Relative colony formation to control	Relative self-renewal to control
Dasatinib	10	3 ± 3	0.76 ± 0.05	0.93 ± 0.06
	50	8 ± 2	0.67 ± 0.09	1.04 ± 0.07
	100	25 ± 6	0.59 ± 0.09	0.84 ± 0.03
	200	52 ± 6	0.23 ± 0.07	0.73 ± 0.06
Ponatinib	5	5 ± 1	1.04 ± 0.06	0.98 ± 0.09
	25	15 ± 4	0.87 ± 0.09	0.93 ± 0.02
	50	28 ± 5	0.64 ± 0.05	0.76 ± 0.03
	100	43 ± 2	0.43 ± 0.06	0.48 ± 0.05

### Stachydrine inhibits multiple RTK-mediated signalling pathways in CML cells

**Figure 5.**
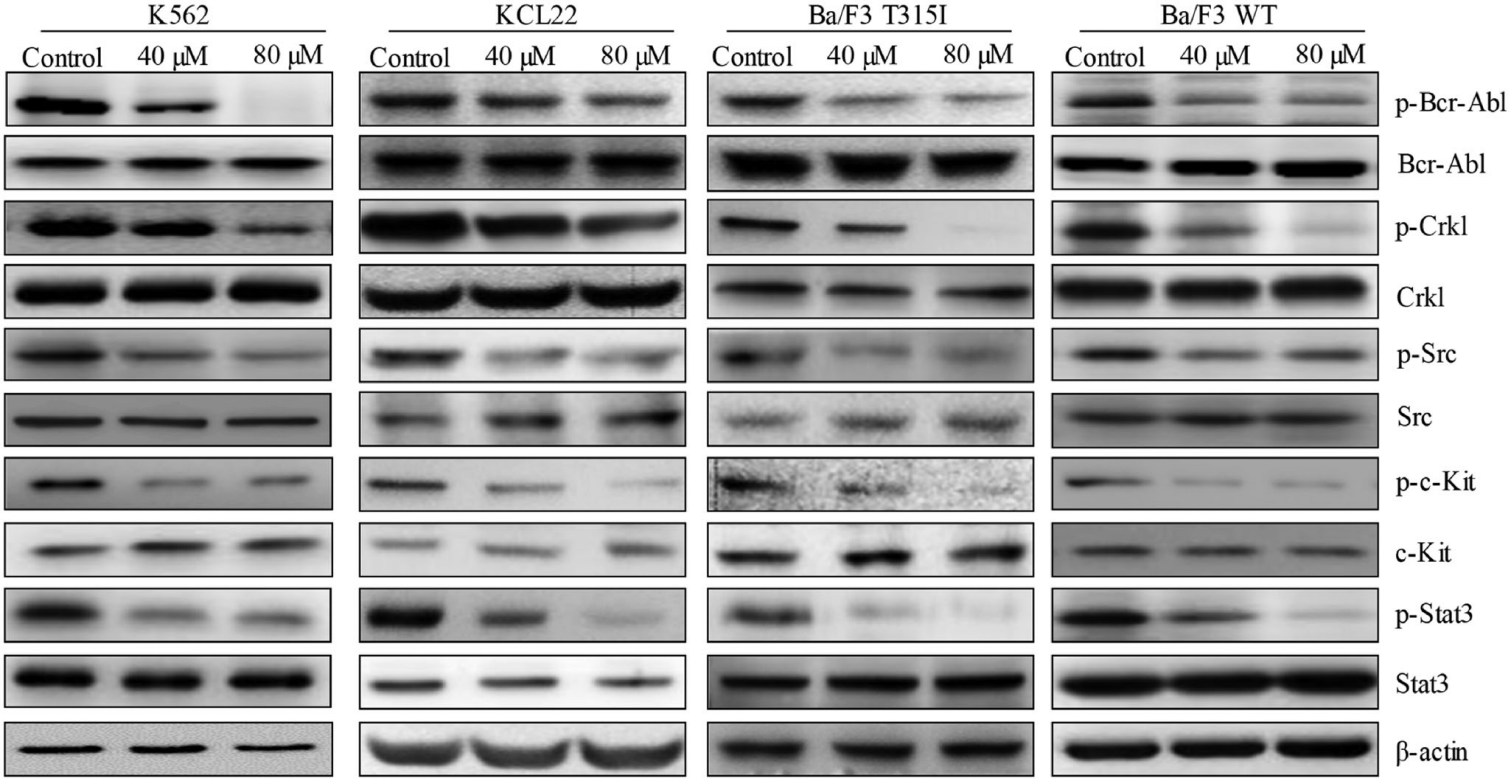
Stachydrine inhibits receptor tyrosine kinases-mediated signalling in CML cells. Representative western blotting images showing the level of phosphor- and total Bcr-Abl, Crkl, Src, c-Kit and Stat3 inK562, KCL22, Ba/F3 WT and Ba/F3 T315I cells exposed to stachydrine.

**Figure 6.**
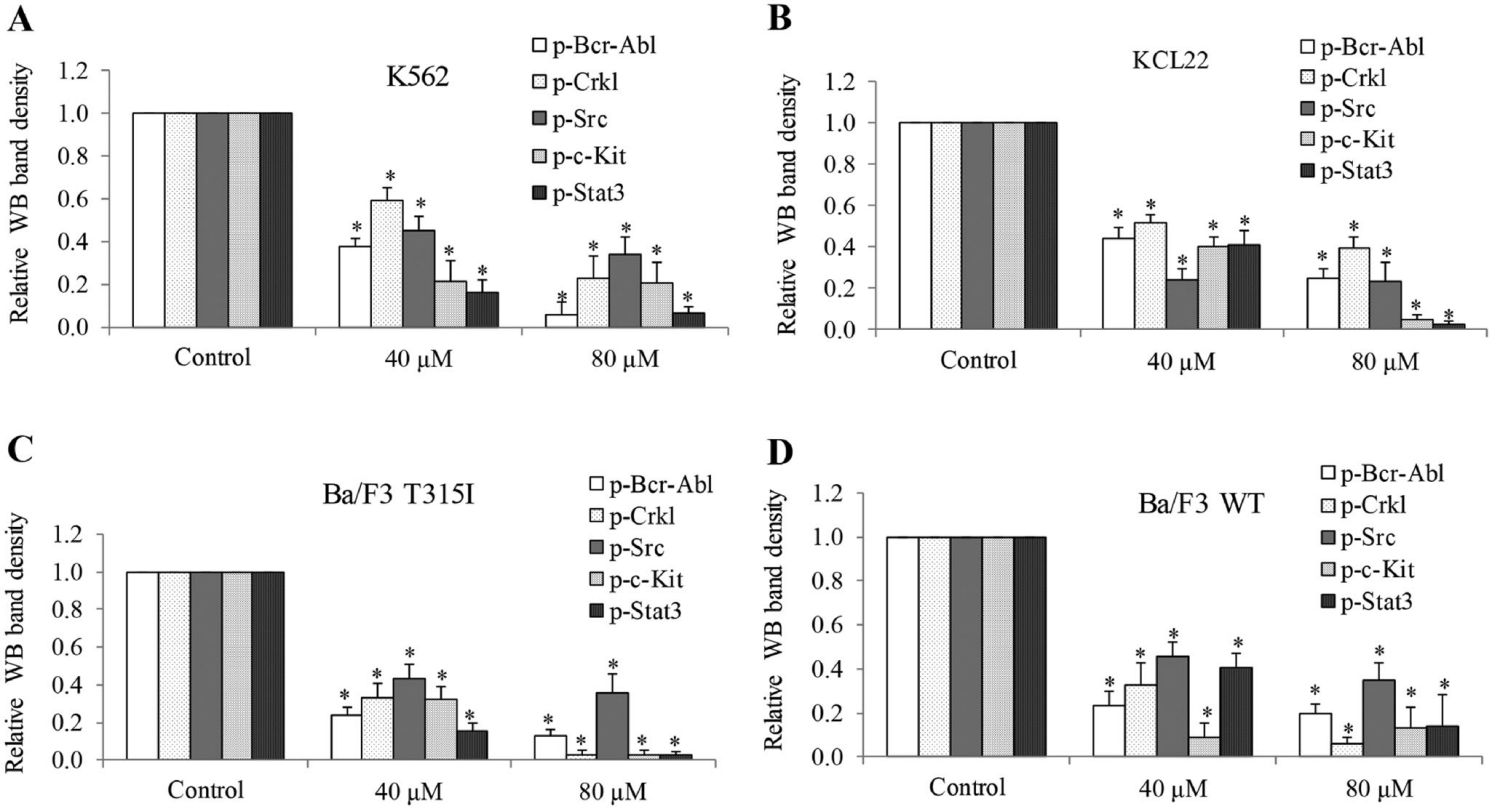
Quantification of western blot band densities using imaging J software showing significant reduction of p-Bcr-Abl, p-Crkl, p-Src and p-c-Kit in K562 (A), KCL22 (B) and Ba/F3 T315I (C) and Ba/F3 WT (D) cells after stachydrine treatment. *p < 0.05, compared to control.

To understand the underlying molecular mechanisms of stachydrine’s action in CML cells, we first examined whether stachydrine affects Bcr-Abl signalling as stachydrine is effective on CML cells regardless of Bcr-Abl mutations. We performed western blotting analysis of phosphor- and total levels of Bcr-Abl and Crkl in CML cells after stachydrine treatment. p-Crkl is a marker to predict the efficacy of TKIs in the treatment of CML patients (La Rosee et al. 2008). We found that stachydrine potently decreased p-Bcr-Abl and p-Crkl but not total level of Bcr-Abl and Crkl in imatinib-sensitive cells (Figures 5 and 6). As expected, we observed the same in imatinib-resistant Ba/F3 T315I cells. It is of interest to note that apart from Bcr-Abl, stachydrine also potently decreased p-Scr and p-c-Kit but not total level of Src and c-Kit (Figures 5 and 6). Decreased phosphorylation of Stat3, a downstream effector of Scr (Bowman et al. 2001), was observed in stachydrine-treated CML cells. Our results demonstrate that stachydrine inhibits activity of multiple RTKs and RTK-mediated signalling pathways in CML. It was noted that the inhibitory effects on RTK-mediated signalling pathways by stachydrine was not limited to KCL22 and Ba/F3 T315I cells; K562 and Ba/F3 WT cells responded in a similar manner (Figures 5 and 6).

**Figure 7.**
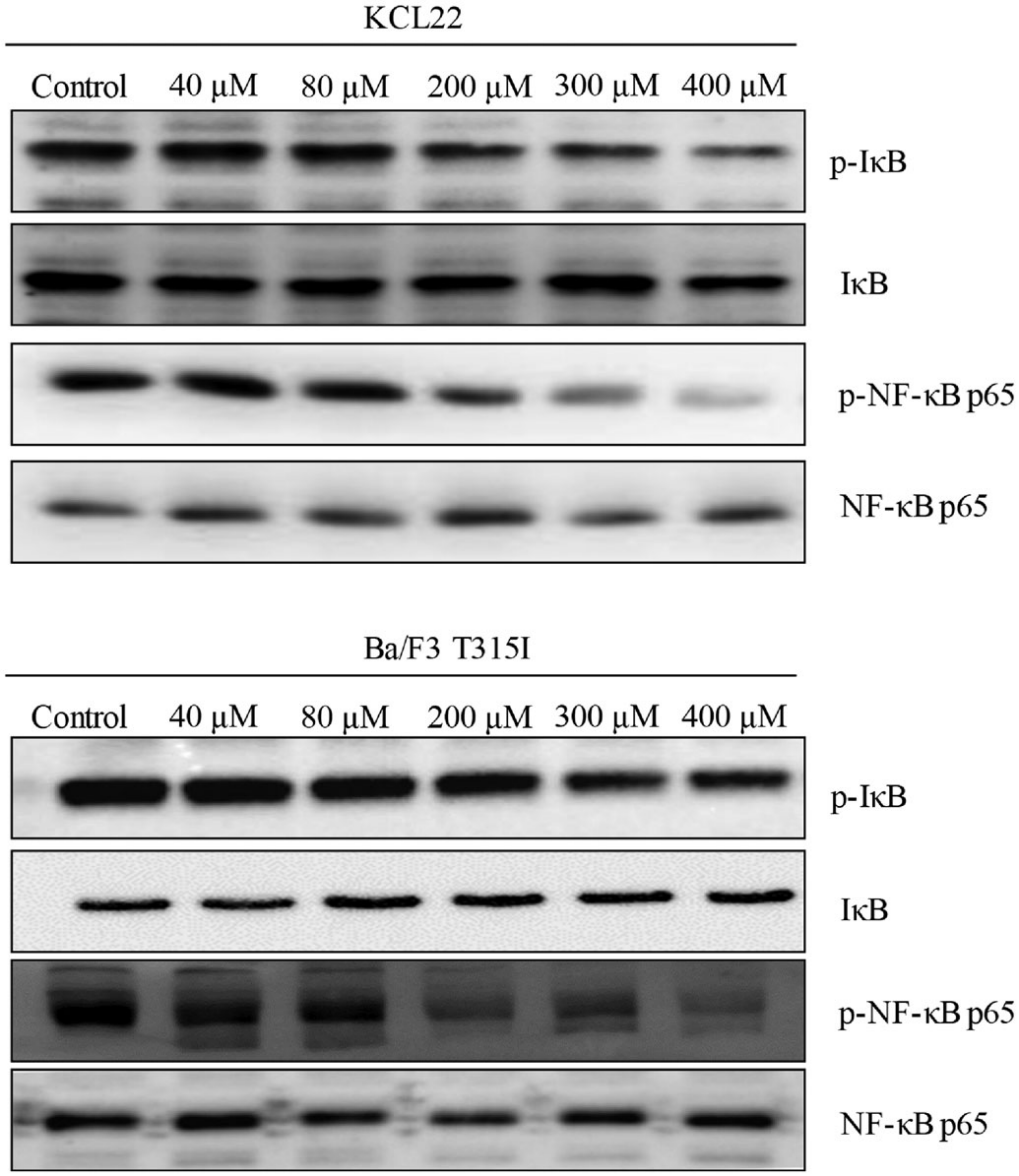
Representative western blotting images showing the level of phosphor- and total IĸB and NF-ĸB p65 in KCL22 and Ba/F3 T315I cells exposed to stachydrine.

Various studies have reported that stachydrine decreases phosphorylation of IĸB at Ser32 and NF-κB at Ser536, leading to inhibition of NF-κB signal pathway (Guo et al. 2012; Chen et al. 2021). As NF-κB signal pathway plays an important role in Bcr-Abl-mediated pathways in CML (Carra et al. 2016), we tested the effects of stachydrine on p-IĸB and p-NF-κB in CML cells after stachydrine treatment. Our results showed that stachydrine at 40 and 80 μM did not affect p-IĸB and p-NF-κB in KCL22 and Ba/F3 T315I cells. Stachydrine at higher concentrations, such as 200 μM and above, did significantly inhibit p-IĸB and p-NF-κB (Figure 7). Since stachydrine at 40 and 80 μM is active against CML cells, our findings suggest that the inhibition of NF-κB signal pathway by stachydrine at 200 μM and above is not attributed to its anti-CML activity.

## Discussion

Although Bcr-Abl TKIs have revolutionized the treatment of CML, they are not effective in BP-CML. Herein, we demonstrate the preclinical activity of stachydrine in CML. Many studies had highlighted that stachydrine displayed anticancer activities in various preclinical cancer models (Rathee et al. 2012; Isozaki et al. 2014; Wang et al. 2017; Liu et al. 2018). Our findings also support that stachydrine has broad inhibitory effects on proliferation, survival, colony formation and self-renewal in CML cell lines and primary BP-CML patient samples, including cells harbouring T315I mutation. In addition, we further demonstrate that stachydrine displays anti-leukaemia selectivity and acts synergistically with Bcr-Abl TKIs, via suppressing activity of RTKs.

Using a panel of CML cell lines that cover varying cellular origins and Bcr-Abl mutations, we demonstrate that stachydrine is active against CML with IC_50_ ranging from 22 μM to 140 μM (Figure 1(A) and Table 1). The IC_50_ of stachydrine in CML is similar to those in a variety of cancers, including prostate cancer, breast cancer and astrocytoma (Rathee et al. 2012; Wang et al. 2017; Liu et al. 2018). Our results together with previous studies suggest that the effective dose of stachydrine in cancer is at micromolar concentration.

The findings that stachydrine inhibits biological properties of BP-CML stem/progenitor cells, such as proliferation, survival, differentiation and self-renewal (Figure 2), are significant. This is because leukaemia stem/progenitor cells represent a reservoir of BP-CML and this is a potential source to initiate relapse, and these cells are resistant to TKIs and many other drugs (Houshmand et al. 2019). It is of interest to note that the inhibitory effects of stachydrine on leukaemia stem cells are irrespective of Bcr-Abl overexpression, mutations and TKI-resistance (Table 2). The T315I mutation is frequently found in BP-CML patients who develop resistance to first- and second-generation TKIs (Hiwase et al. 2011; Nicolini et al. 2013). Our findings clearly demonstrate that stachydrine is effective in targeting T315I cells in a similar manner as WT cells (Figures 1 and 2). The combination of stachydrine and TKIs is synergistic for both CML cell lines and BP-CML stem/progenitor cells (Figures 3 and 4), suggesting the potential of stachydrine to overcome TKI resistance in CML. We also observed the selective anti-leukaemia activity of stachydrine (Figures 2 and 4). This is consistent with previous work that stachydrine at effective concentration against astrocytoma cells did not produce significant cytotoxicity in normal human astrocytes (Liu et al. 2018).

Identification of agents that target differentiated and stem/progenitor cells is a potential strategy for cancer treatment. Compared to previous studies on the efficacy of stachydrine against cancer, our study is the first to demonstrate (1) the anticancer activity on primary patient cancer cells; (2) the inhibitory effects on cancer stem cells; (3) the synergism between stachydrine and other anticancer drugs. Future *in vivo* studies using CML patient-derived xenograft mouse model will further investigate the benefit of stachydrine as a therapeutic option in CML via evaluating the effective dose, toxicity and pharmacokinetic profiling.

We further examined and bring clarity to the existing known mechanisms of action of stachydrine in cancer. Stachydrine acts on CML via inhibiting multiple RTKs’ activity as shown in our findings. It decreased p-Bcr-Abl, p-c-Kit and p-Src in CML WT and T315I cells (Figures 5 and 6). Apart from Bcr-Abl, c-Kit and Src are oncogenic RTKs that are very important for leukaemia growth and survival (Agarwal et al. 2013; Soverini et al. 2018). The ability of stachydrine in targeting multiple RTKs makes it advantageous over Bcrl-Abl TKI. We previously identified that gallic acid, a plant extract from TCM, is effective against acute myeloid leukaemia (Gu et al. 2018). It is of interest to note that gallic acid also inhibits Bcr-Abl and Src signalling pathways in leukaemia and lung cancer (Chandramohan Reddy et al. 2012; Phan et al. 2016). Stachydrine and gallic acid are bioactive component of TCM. Increasing evidence has shown the various therapeutic activities of TCM in cancer (Xiang et al. 2019). Both our previous and current work suggests that inhibiting RTK might be one of the underlying mechanisms of TCM’s anticancer activities.

## Conclusions

Our work provides pre-clinical evidence that combined treatment of stachydrine and TKIs represents an alternative therapeutic strategy for BP-CML patients. Our work also highlights the inhibitory effects of stachydrine on various RTKs in cancer cells.

## Supplementary Material

Supplemental Material
