# Supplementary material for: Stachydrine is effective and selective against blast phase chronic myeloid leukaemia through inhibition of multiple receptor tyrosine kinases
Source: Pharm Biol. 2022 Mar 29;60(1):700–7. doi: 10.1080/13880209.2022.2044862 (PMC8967197; doi:10.1080/13880209.2022.2044862)
Supplement: Supplemental Material [file IPHB_A_2044862_SM4332.doc]

**Stachydrine is effective and selective against blast phase chronic myeloid leukemia through inhibition of multiple** **receptor tyrosine kinases**

**Fig. S1.** Representative flow cytometry plots showing the percentage of Annexin V and 7-AAD staining in multiple CML cell lines treated with stachydrine.

**Fig. S2.** Representative colony formation images in BP-CML and CB CD34+ cells treated with stachydrine.

**Fig. S3.** Representative flow cytometry plots showing the percentage of Annexin V and 7-AAD staining in CB and BP-CML CD34 cells treated with stachydrine.
